# US soldiers and the role of leadership: COVID-19, mental health, and adherence to public health guidelines

**DOI:** 10.1186/s12889-022-13345-z

**Published:** 2022-05-11

**Authors:** Amy B. Adler, Ian A. Gutierrez, Stephanie A. Q. Gomez, Matthew R. Beymer, Theresa Jackson Santo, Jeffrey L. Thomas, David S. Cates, Amy Millikan Bell, Phillip J. Quartana

**Affiliations:** 1 Walter Reed Army Institute of Research, 503 Robert Grant Ave, Silver Spring, MD 20910 USA; 2 US Army Public Health Center, Aberdeen Proving Ground, Aberdeen, MD USA; 3 National Defense University, Washington, DC USA; 4 Nebraska Medicine, University of Nebraska Medical Center, Omaha, NE USA

**Keywords:** Pandemic, Health promotion, Supervisor, Military, Occupational health

## Abstract

**Background:**

Previous studies have documented the impact of domain-specific leadership behaviors on targeted health outcomes in employees. The goal of the present study was to determine the association between specific leadership behaviors addressing COVID-19 and US soldiers’ mental health and adherence to COVID-19 public health guidelines.

**Methods:**

An electronic, anonymous survey was administered to US Army soldiers across three major commands (*N* = 7,829) from December 2020 to January 2021. The primary predictor of interest was soldiers’ ratings of their immediate supervisors’ behaviors related to COVID-19. The outcomes were soldiers’ mental health (i.e., depression and generalized anxiety) and adherence to COVID-19 public health guidelines. Covariates were rank, gender, ratings of immediate supervisors’ general leadership, level of COVID-19 concerns, and COVID-19 status (e.g., tested positive, became seriously ill). Logistic regressions were used to model the unique association of COVID-19 leadership behaviors with outcomes after adjusting for covariates.

**Results:**

High levels of COVID-19 leadership behaviors were associated with lesser likelihood of soldiers’ screening positive for depression (AOR = 0.46; 95% CI [0.39, 0.54]) and anxiety (AOR = 0.54; 95% CI [0.45, 0.64]), and greater likelihood of frequent adherence to preventive health guidelines (AORs = 1.58; 95% CI [1.39, 1.80] to 2.50; 95% CI [2.01, 3.11]).

**Conclusion:**

Higher levels of COVID-19 leadership behaviors may support soldiers’ mental health and encourage their adherence to COVID-19 public health guidelines. Given the link between these leader behaviors and soldier adaptation to the pandemic over and above general leadership, training for supervisors should focus on targeting specific health-promoting behaviors. Results can inform leader training for the military and other high-risk occupations.

Millions of Americans have contracted the novel coronavirus (COVID-19), and hundreds of thousands have died since the start of the pandemic [1]. Daily life has been disrupted in a variety of ways, from restrictions on businesses to remote learning for school children [2]. Following public health guidelines such as mask wearing and physical distancing is related to lower rates of COVID-19 infection [3], and studies with diverse samples provide a range of estimates regarding adherence to these public health recommendations [4, 5]. There is also some evidence suggesting an increase in anxiety and depression in the civilian population since the onset of the pandemic [6, 7], and there are also concerns about the impact of the pandemic on military service members [8, 9]. While military personnel are in a unique position with respect to job security, stable housing, and free personal and family medical care, service members remain susceptible to the myriad stressors that civilians experience during the pandemic; military personnel may still experience stress related to concern that they or their family members could contract COVID-19, the loss of income from civilian jobs contributing to household income, and the strain of social distancing requirements.

For these reasons, it is important to identify occupationally-relevant factors that can help mitigate the impact of pandemic-related stress on service members’ mental health and day-to-day engagement in health-related behaviors. One such factor is the influence of supervisory leadership. Research has documented an association between general leadership attributes and better health-related outcomes in civilians and service members [10–12]. While general leadership, exemplified by qualities such as effectiveness, is a valuable predictor of these outcomes, its broad characterization does not provide specific and practical guidance for immediate supervisors [13, 14]. Although studies have found negative employee outcomes associated with certain types of leadership during the pandemic [15], pre-pandemic studies that have examined specific leadership behaviors in the domains of personnel safety [13], family supportive behaviors [16], and sleep [17], have shown beneficial health and occupational outcomes for employees above and beyond general leadership.

Building on these findings regarding domain-specific leadership, we focused on the domain of health-promoting leadership to address COVID-19 concerns. Previous studies have found that health-promoting leadership behaviors were associated with less emotional exhaustion in service members deployed to Afghanistan on a medical mission [18], and with more positive attitudes toward quarantine in service members returning from deployment to west Africa in response to the Ebola outbreak of 2014 [19]. To assess the role of these health-promoting leadership behaviors in the context of the pandemic, we adapted items to develop a scale of COVID-19 leadership behaviors, and administered these items in a cross-sectional survey of U.S. Army soldiers.

The survey included two outcomes that are important for individual health and organizational functioning. First, we examined measures of depression and anxiety in light of their prevalence in the military population[20] as well as civilian research documenting an increase of these disorders during the pandemic [6]. Second, we examined soldiers’ adherence to COVID-19 public health guidelines designed to prevent the spread of COVID-19, including hand washing, mask wearing, and avoiding large gatherings. We also used a measure of COVID-19 leadership behaviors to assess the relationship between leader engagement in these behaviors and study outcomes after controlling for general leadership and other covariates. This study offers a unique opportunity to examine the relationship between COVID-19 leadership behaviors and both mental health and compliance with pandemic-related public health guidance in the context of a high-risk occupation like the military.

## Methods

### Study population

The Walter Reed Army Institute of Research (WRAIR) and the Army Public Health Center (APHC) Behavioral Health Advisory Team (BHAT) invited all U.S. Army soldiers across three major commands from December 9th, 2020 to January 19th, 2021 to participate in the survey. This data collection occurred prior to the U.S. Food and Drug Administration’s emergency use authorization of COVID-19 vaccines. Soldiers were invited to participate via a link sent through military communication channels as part of routine operations. The survey included an information page about participation and a screener to determine eligibility. Respondents were allowed to proceed to the full survey if they agreed to participate and were then asked if they consented to let their data be used for research purposes. Survey participation was voluntary, and soldiers were not compensated for participation. This survey was approved by the WRAIR human research protection branch and the APHC office of human protections.

### Inclusion criteria

Soldiers assigned to the three Army commands participating in the survey were eligible for inclusion. Respondents were active-duty military or activated reservists. Civilians and contractors were not eligible to complete the survey.

### Measures

#### Mental health

Mental health was measured by screening for depression and anxiety. Depression symptoms were measured with the two-item Patient Health Questionnaire (PHQ-2) [21]. Anxiety symptoms were measured with the two-item Generalized Anxiety Disorder (GAD) scale [22]. For both measures, soldiers rated each item on a 4-point scale ranging from 0 (*Not at all*) to 3 (*Nearly every day*). These items were followed by a question on functional impairment (“How difficult have these problems made it for you to do your work, take care of things at home, or get along with other people?”) [23]; this item was rated on a 4-point scale ranging from 0 (*Not difficult at all*) to 3 (*Extremely difficult*). For both depression and anxiety, scores of 3 or higher accompanied by reports of any functional impairment (scores of 1 or more) were regarded as a positive screen.

#### Adherence to COVID-19 public health guidelines

Adherence to COVID-19 public health guidelines was assessed with eight items (e.g., wearing a mask or face covering; coughing or sneezing into your elbow or using a tissue) developed for this survey. Soldiers reported their frequency of engaging in each behavior using a 5-point scale ranging from 1 (*Never*) to 5 (*Always*). Reliability was not calculated because these items were treated as separate outcomes rather than as a scale. High adherence to each public health guideline was defined as a score of 4 or 5 (frequently or always) and low adherence to each public health guideline was defined as a score of 3 or lower.

#### COVID-19 leadership behaviors

COVID-19 specific leadership behaviors were assessed with 14 items from the health-promoting leadership scale[18, 19] adapted based on an intervention to promote resilience during facility-based quarantine after exposure to COVID-19 [24]. Soldiers rated their immediate supervisor on each item using a 5-point scale ranging from 1 (*Strongly disagree*) to 5 (*Strongly agree*). High scores on the COVID-19 leadership behaviors scale were defined as a mean of 3.5 or higher (i.e., a mean score rounding toward “agree” or higher), and low scores were defined as lower than 3.5. This cutoff reflects a conceptually meaningful difference between low and high categories. Internal consistency was high (Cronbach’s alpha = 0.97).

### Covariates

#### Rank

Given the established relationship between rank and mental health and health-related behaviors [25, 26], we accounted for rank in our analyses. Military rank was classified into three groups: (1) junior enlisted soldiers (E1-E4), (2) non-commissioned officers (E5-E9), and (3) officers (O1-O9) and warrant officers (WO1-WO5).

#### Gender

Given the potential effect of gender on mental health and health-related behaviors [25, 27], we accounted for gender in our analyses. Gender was classified into three groups: male, female and prefer not to respond.

#### General leadership

General leadership was included as a covariate to ensure that the relationships between COVID-19 leadership behaviors and study outcomes were not better explained by the quality of leadership in general. General leadership was measured with the five-item Perceived Leader Effectiveness scale [28]. Soldiers rated each item (e.g., “My immediate supervisor is an effective leader” and “My immediate supervisor displays strong leadership abilities”) on a 5-point scale ranging from 1 (*Strongly disagree*) to 5 (*Strongly agree*). High scores on the general leadership scale were defined as a mean of 3.5 or higher (i.e., a mean score rounding toward “agree” or higher), and low scores were defined as a mean score of lower than 3.5. This cutoff reflects a conceptually meaningful difference between low and high categories. Internal consistency was high (Cronbach’s alpha = 0.96).

#### COVID-19 concerns

COVID-19 concerns were included as a covariate to ensure that COVID-19 leadership behaviors explained outcomes over and above worries related to the pandemic. COVID-19 concerns were measured with 20 items developed for this survey. For each item, participants rated the extent to which they were worried or concerned about a range of factors related to COVID-19 such as accessing medical care, engaging in social activities, and the changing rules, regulations and guidance related to COVID-19. Soldiers rated each item on a 5-point scale ranging from 0 (*Not at all*) to 4 (*Extremely*). High levels of COVID-19 concerns were defined as a mean score of 1.5 or higher (i.e., more than “slightly” concerned on average), and low levels of COVID-19 concern were defined as a mean score of lower than 1.5. This cutoff was chosen to reflect meaningful differences in average level of concern related to COVID-19. Internal consistency was high (Cronbach’s alpha = 0.94).

#### COVID-19 status

COVID-19 status was included as a covariate to ensure that COVID-19 leadership behaviors explained outcomes over and above COVID-19 infection and/or illness given the potential link between COVID-19 status and mental health symptoms [29, 30]. COVID-19 status was measured with five items developed for this survey. Soldiers were asked if they had tested positive for the virus, been diagnosed with COVID-19 by a medical professional, become seriously ill with COVID-19, been hospitalized with COVID-19, or recovered from COVID-19. Soldiers who responded “no” to all items were deemed COVID-19 negative, while those who responded “yes” to any one item were deemed COVID-19 positive.

### Statistical analysis

Among survey participants (*n* = 7,829), each mental health item had no more than 11.7% missing, and each item assessing adherence to COVID-19 public health guidelines had no more than 7.0% missing. Each item on the COVID-19 leadership behaviors scale had no more than 11.4% missing, and all other model predictors had no more than 11.1% missing. Multivariable logistic regression models removed missing data using listwise deletion. Frequencies were calculated for the outcomes of interest (positive mental health screens and adherence to COVID-19 public health guidelines) as well as COVID-19 leadership behaviors. Unadjusted and adjusted multivariable logistic regression models were calculated. All analyses were conducted in R v.4.1.0 [31].

## Results

Of the approximately 72,000 soldiers assigned to the units that were surveyed, 11,340 responded to the survey link and passed the screener for eligibility. Of these participating soldiers, 69.0% (*n* = 7,829) provided consent to have their responses used for research purposes. Among valid responders, 83.4% (*n* = 6,337) were male, 63.0% (*n* = 3,036) were less than 30 years of age, 59.0% (*n* = 3,663) identified as non-Hispanic White; 66.7% (*n* = 5,079) had some college education, 53.0% (*n* = 3,996) were married, and 47.2% (*n* = 3,580) were junior enlisted. These demographic frequencies are comparable to those of the Army as a whole [32], although our sample was slightly more racially diverse compared to Army-wide estimates (41.0% vs. 32.4%, respectively [32]. With respect to COVID-19 status, 8.5% (*n* = 668) endorsed at least one COVID-19 status item, with 5.3% (n = 412) reporting they had tested positive for the virus.

Frequencies for mental health and adherence to COVID-19 public health guidelines are presented in Table 1. Regarding mental health, 16.8% (*n* = 1,168) screened positive for anxiety, and 17.4% (*n* = 1,206) screened positive for depression. A majority of soldiers reported frequently adhering to COVID-19 public health guidelines; soldiers were most likely to report frequently or always wearing a mask or face covering (89.2%; *n* = 6,508), while they were least likely to report frequently or always staying home (54.2%; *n* = 3,950).

**Table 1:** Frequencies of Positive Mental Health Screens and Adherence to Public Health Guidelines

	n/N (%)
	**Positive Screen**
*Mental Health*	
Anxiety with Impairment	1,168/6,936 (16.8%)
Depression with Impairment	1,206/6,933 (17.4%)
	**Frequently or Always**
*Adherence to Public Health Guidelines*	
Wearing a mask or face covering	6,508/7,297 (89.2%)
Coughing/sneezing into your elbow or use a tissue	6,374/7,290 (87.4%)
Washing your hands frequently for 20 s with soap and water	5,888/7,296 (80.7%)
Using hand sanitizer when you can’t wash your hands	5,758/7,296 (78.9%)
Avoiding people with COVID-19 symptoms	5,167/7,308 (70.7%)
Monitoring yourself for fever, coughing, or shortness of breath	5,082/7,279 (69.8%)
Avoiding non-work related indoor gatherings	4,586/7,297 (62.8%)
Staying at home	3,950/7,294 (54.2%)

*n* = number screening positive or adhering to public health guidelines. N = number responding to mental health screen or public health survey item

With respect to leadership, 64.5% (*n* = 4,512) of soldiers indicated that their immediate supervisor displayed a high level of general leadership, and 59.5% (*n* = 4,169) indicated that their immediate supervisor engaged in high levels of COVID-19 leadership behaviors. The percent of soldiers agreeing or strongly agreeing that their immediate supervisor engaged in each COVID-19 leadership behavior is provided in Table 2. For all but one item, a majority of soldiers agreed or strongly agreed that their immediate supervisor engaged in the target COVID-19 leadership behavior; soldiers were most likely to agree that their immediate supervisor encouraged them to report any symptoms of COVID-19 (72.2%; *n* = 5,035), whereas soldiers were least likely to agree that their immediate supervisor talked to them about the way that COVID-19 had personally impacted them (41.7%; *n* = 2,904).

**Table 2:** Frequencies of COVID-19 Leadership Behaviors

	Agree or Strongly Agree
	n/N (%)
Encourages us to report any symptoms of COVID-19 we might have	5,035/6,976 (72.2%)
Leads by example by following health guidelines to reduce the spread of COVID-19 (such as social distancing, handwashing, using mask/face covering)	4,700/6,969 (67.4%)
Has shared useful and accurate information about the COVID-19 pandemic	4,511/6,974 (64.7%)
Provides updates about recent COVID-19 pandemic related developments	4,443/6,960 (63.8%)
Takes steps to keep us socially connected as a unit during the COVID-19 pandemic	4,320/6,975 (61.9%)
Acknowledges the stress of uncertainty related to the COVID-19 pandemic	4,252/6,940 (61.3%)
Emphasizes taking care of ourselves mentally during the COVID-19 pandemic	4,206/6,959 (60.4%)
Encourages us to think positively during this COVID-19 pandemic	4,123/6,973 (59.1%)
Reminds Soldiers during the COVID-19 pandemic that we are here to serve with honor, serve a mission, and serve a greater purpose	3,969/6,948 (57.1%)
Has modified unit tasks to prevent Soldiers from working in close proximity to one another	3,890/6,962 (55.9%)
Ensures we have basic supplies for daily living (like food, soap and toilet paper) during the COVID-19 pandemic	3,848/6,971 (55.2%)
Focuses on what to be grateful for during the COVID-19 pandemic	3,612/6,972 (51.8%)
Encourages us to identify what we can and cannot control about COVID-19 pandemic	3,487/6,965 (50.1%)
Talks about the way the COVID-19 pandemic is personally impacting them	2,904/6,972 (41.7%)

*n* = number of Agree or Strongly Agree responses. N = valid number responding to survey item

**Fig. 1:**
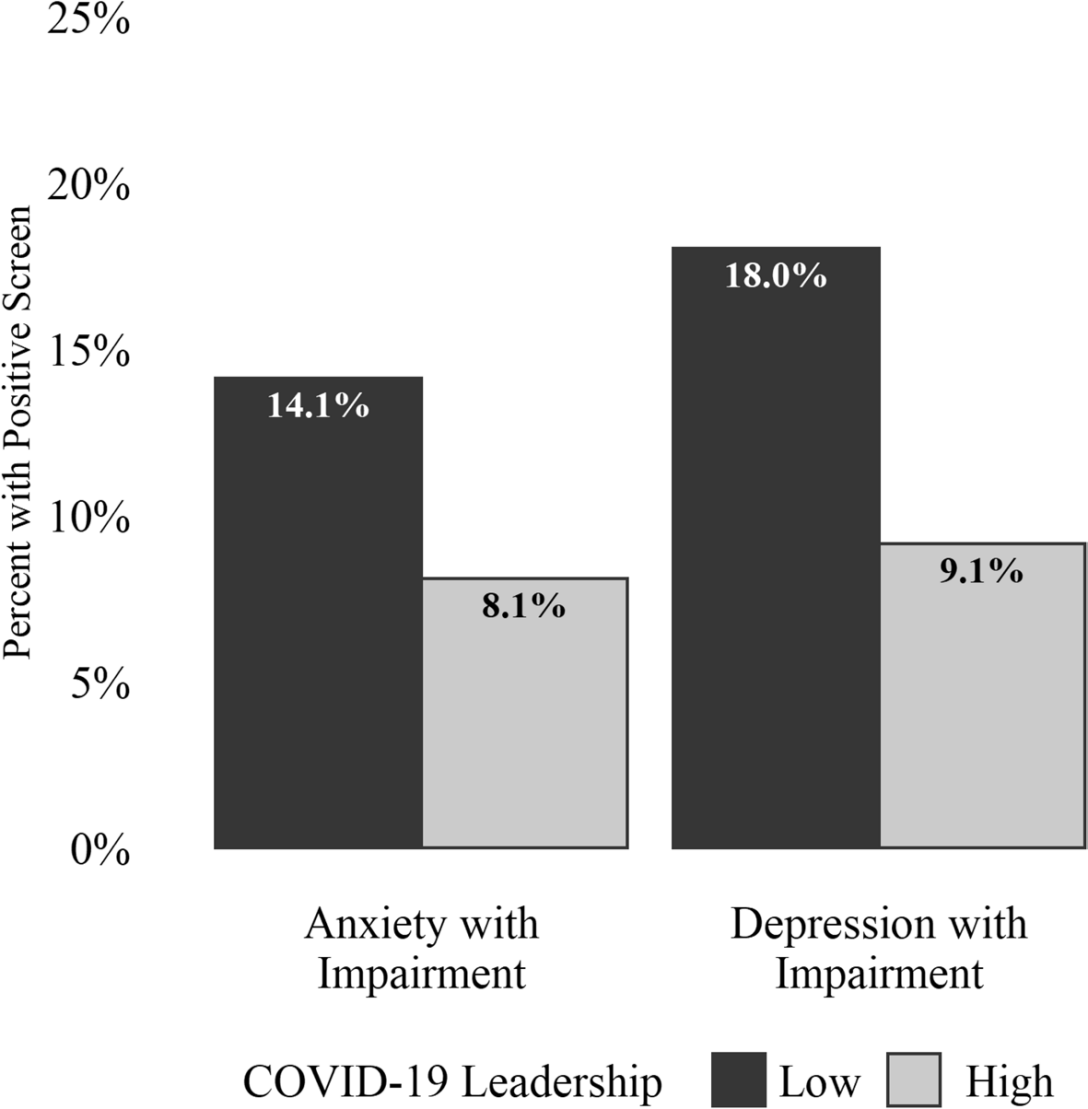
Percent positive screens for depression and anxiety by level of immediate supervisor’s COVID-19 leadership adjusted for rank, gender, general leadership, COVID-19 concern, and COVID-19 status. To illustrate these relationships, adjusted rates of positive screens are presented for junior enlisted rank, male gender, low general leadership, low levels of COVID-19 concern, and COVID-19 negative status

Table 3 provides unadjusted and adjusted odds ratios for screening positive for depression and anxiety and soldier ratings of their immediate supervisor’s COVID-19 leadership behaviors, with adjusted odds ratios (AORs) controlling for rank, gender, general leadership, COVID-19 concerns, and COVID-19 status. COVID-19 leadership behaviors were inversely associated with screening positive for depression (AOR = 0.46; 95% CI [0.39, 0.54]) and anxiety (AOR = 0.54; 95% CI [0.45, 0.64]). Adjusted estimates of prevalence of positive screens for depression and anxiety are presented in Fig. 1.

**Table 3:** Odds ratios for anxiety, depression, and adherence to public health guidelines by high level of COVID-19 leadership

	Odds Ratio (95% CI)	Adjusted Odds Ratio^1^ (95% CI)
*Mental Health*		
Anxiety with Impairment (Positive Screen)	0.43 (0.38, 0.48)*	0.54 (0.45, 0.64)*
Depression with Impairment (Positive Screen)	0.36 (0.32, 0.41)*	0.46 (0.39, 0.54)*
*COVID-19 Public Health Guidelines*		
Wearing a mask or face covering	3.83 (3.26, 4.51)*	2.50 (2.01, 3.11)*
Coughing/sneezing into your elbow or use a tissue	3.16 (2.73, 3.67)*	1.85 (1.51, 2.27)*
Washing your hands frequently for 20 s with soap and water	2.70 (2.40, 3.06)*	2.31 (1.96, 2.71)*
Using hand sanitizer when you can’t wash your hands	2.69 (2.39, 3.03)*	2.29 (1.96, 2.69)*
Monitoring yourself for fever, coughing, or shortness of breath	2.57 (2.31, 2.86)*	2.17 (1.88, 2.50)*
Avoiding people with COVID-19 symptoms	2.17 (1.96, 2.41)*	1.91 (1.66, 2.20)*
Avoiding non-work related indoor gatherings	2.01 (1.82, 2.22)*	1.83 (1.60, 2.09)*
Staying at home	1.58 (1.44, 1.74)*	1.58 (1.39, 1.80)*

Mental health and adherence to public health guidelines are predicted by soldier reports of their immediate supervisors engaging in high (vs. low) levels of COVID-19 leadership behaviors

^1^Adjusted for rank category (junior enlisted, non-commissioned officers, or officer/warrant officer), gender (male, female, or prefer not to respond), general leadership, COVID-19 concerns (high versus low), and COVID-19 status. *p < 0.001

**Fig. 2:**
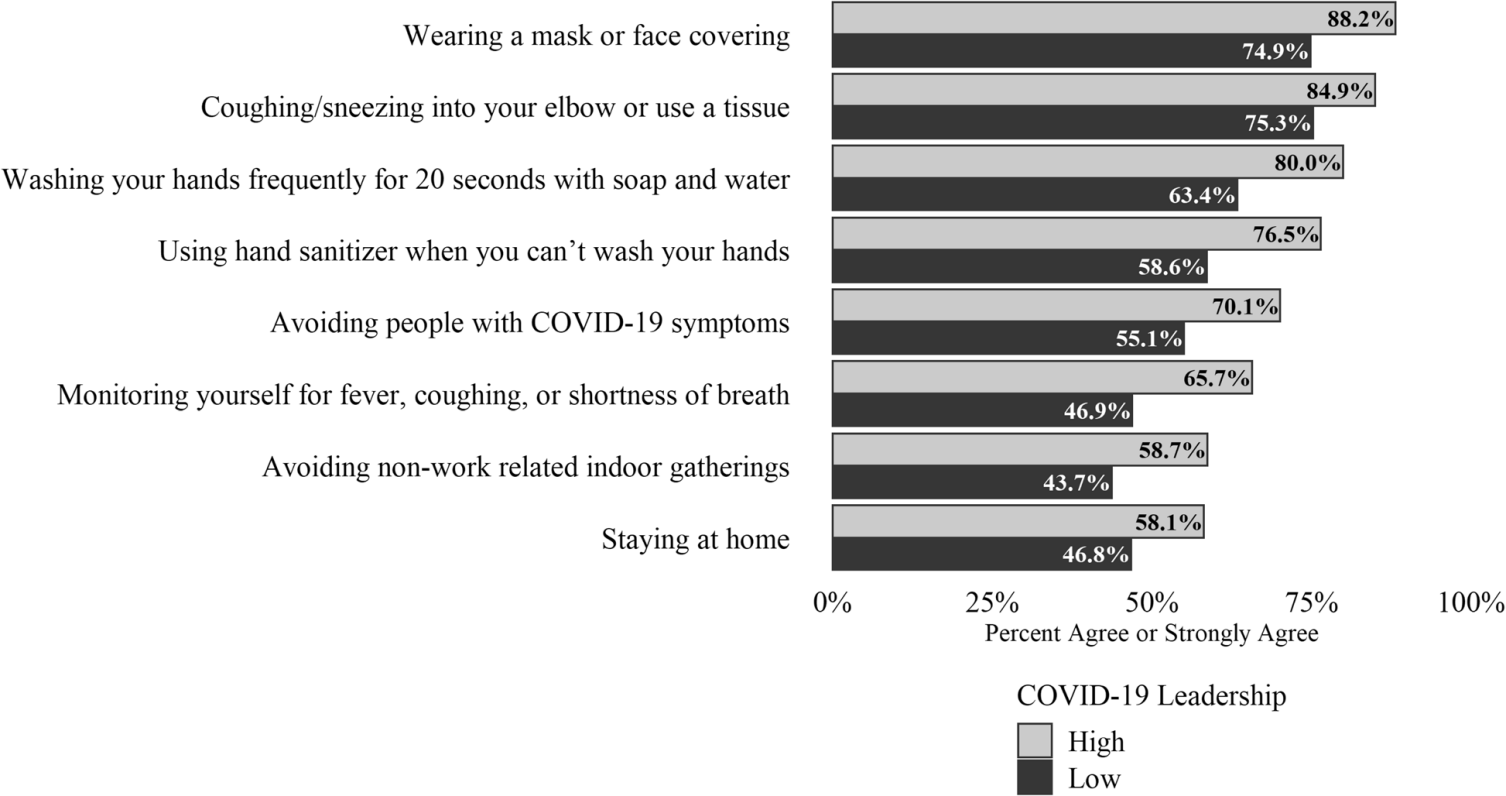
Percent agreement for adherence to public health guidelines by level of immediate supervisor’s COVID-19 leadership adjusted for rank, gender, general leadership, COVID-19 concern, and COVID-19 status. To illustrate these relationships, adjusted rates of adherence to public health guidelines are presented for junior enlisted rank, male gender, low general leadership, low levels of COVID-19 concern, and COVID-19 negative status

Table 3 also provides unadjusted and adjusted odds ratios for adhering to public health guidelines and soldier ratings of their immediate supervisor’s COVID-19 leadership behaviors, with AORs controlling for rank, general leadership, COVID-19 concerns, and COVID-19 status. COVID-19 leadership behaviors significantly predicted each item assessing adherence to COVID-19 public health guidelines after accounting for covariates. COVID-19 leadership behaviors were least strongly associated with staying at home (AORs = 1.58; 95% CI [1.39, 1.80]) and most strongly associated with wearing a mask or face covering (AOR = 2.50; 95% CI [2.01, 3.11]). Predicted prevalence of adherence to COVID-19 public health guidelines are presented in Fig. 2.

## Discussion

In this survey of more than 7,800 US soldiers, high levels of COVID-19 leadership behaviors, as measured by soldier ratings of their immediate supervisor, were associated with better mental health and more frequent adherence to COVID-19 public health guidelines among soldiers, even when controlling for general leadership and other covariates. These relationships were meaningful and robust. When soldiers reported that their immediate supervisors engaged in COVID-19 leadership behaviors, the likelihood of screening positive for depression and anxiety was reduced by approximately half, and the likelihood of reporting adherence to COVID-19 public health guidelines increased for each behavior by more than 50%. Notably, the prevalence of anxiety and depression in this sample was approximately comparable to a 2019 anonymous survey administered at a large Army installation using a similar measure [33]. Specifically, anxiety in the present sample was 16.8% compared to 15.2% in the 2019 sample and 17.4% for depression compared to 15.2% in the 2019 sample [33].

Identifying specific steps that the military can take to help service members reduce their risk of depression and anxiety, and increase their adherence to COVID-19 public health guidelines, is critical for service member health and wellbeing as well as reducing disease burden for the organization [3]. The results of the present study highlight one area that the military can target for intervention: leadership behaviors. Importantly, approximately half of soldiers reported that their immediate supervisors engaged in a range of COVID-19 leadership behaviors, which included setting an example, ensuring essential needs are met, sharing information about COVID-19, and adjusting the workplace context to manage the threat of infection. By demonstrating that these leader behaviors are feasible, our results can be used to focus training efforts. The military could, for example, train immediate supervisors to engage in behaviors that support health promotion. Indeed, previous studies have found that even one hour of leadership training on health-related behaviors can positively impact unit members over time [17].

While our data enabled us to model the relationship between COVID-19 leadership behaviors and soldier adaptation to the pandemic, there are some methodological limitations. First, the data were cross-sectional, which prevents any definitive conclusions regarding causality. Second, self-reported survey data may be influenced by recall and reporting biases such as the halo effect in rating leaders and social desirability in reporting adherence. Third, there could be biases in the sample of individuals opting to complete the survey, although the large sample size and the fact that it generally reflects demographic characteristics of the Army at large suggest that that this sample may be representative.

In developing their health communication strategy, organizations like the military should consider the role of leaders, unit climate and individual differences in beliefs about health [34]. Recent studies have found that individual differences, such as trait reactance, perceived injunctive norms, and COVID-19 conspiracy beliefs, are associated with lower likelihood of engaging in preventive health behaviors [34–37]. While providing individuals with clear rules and accurate information is valuable, public-health messaging driven by data alone risks neglecting how health beliefs factor into health behaviors. Therefore, it may also be helpful for leaders to learn motivational interviewing techniques in order to communicate more effectively with individuals serving on their teams [34].

Given the duration and severity of the pandemic, its impact on mental health, and the importance of following public health guidelines, results from the present study provide a pathway for the military and other high-risk occupations to tailor training of supervisors to support team members. Indeed, the leadership findings reported here were integrated into a fact sheet that was rapidly disseminated by military leaders, suggesting that this guidance addressed a gap in support for organizational stakeholders. These results also underscore the value of organizations providing support to individuals in the workplace by developing leader training, consistent with calls for a wider focus on workplace interventions [38].

First responders, health professionals, and others serving in high-risk occupations already reeling from the demands of the pandemic stand to benefit from supervisors who engage in these health-promoting leadership behaviors. Not only may these behaviors prove useful for the current health crisis, but they may also offer a road map for how supervisors can support employees during other periods of stress in which health and wellbeing are seriously threatened. Future research should examine the degree to which these targeted leadership behaviors are associated with adjustment in other high-risk occupational contexts and the degree to which training in these behaviors can promote healthier adaptation.

## Data Availability

The datasets generated during the current study are not publicly available due to institutional regulations protecting service member survey responses but are available from the corresponding author on reasonable request (may require data use agreements to be developed).

## Abbreviations

APHC: Army Public Health Center
AOR: Adjusted Odds Ratio
BHAT: Behavioral Health Advisory Team
CI: Confidence Interval
GAD: Generalized Anxiety Disorder
PHQ-2: Patient Health Questionnaire-2
WRAIR: Walter Reed Army Institute of Research

## References

[1] Centers for Disease Control and Prevention (2021). Estimated COVID-19 Burden.

[2] Chirico F (2021). Coronavirus disease 2019: the second wave in Italy. J Health Res.

[3] Lennon RP (2021). Lower intent to comply with COVID-19 public health recommendations correlates to higher disease burden in following 30 days. South Med J.

[4] Block R (2020). African American Adherence to COVID-19 Public Health Recommendations. Health Lit Res Pract.

[5] Lennon RP (2020). Public Intent to Comply with COVID-19 Public Health Recommendations. Health Lit Res Pract.

[6] Vahratian A (2021). Symptoms of Anxiety or Depressive Disorder and Use of Mental Health Care Among Adults During the COVID-19 Pandemic - United States, August 2020-February 2021. MMWR Morb Mortal Wkly Rep.

[7] Ettman CK (2020). Prevalence of Depression Symptoms in US Adults Before and During the COVID-19 Pandemic. JAMA Netw Open.

[8] Han RH (2020). Planning for Mental Health Needs During COVID-19. Curr Psychiatry Rep.

[9] Wynn G (2020). Military mental health and COVID-19. Journal of Military, Veteran and Family Health.

[10] Jones N (2012). Leadership, cohesion, morale, and the mental health of UK Armed Forces in Afghanistan. Psychiatry.

[11] Lopez AA (2019). Validation of the WRAIR Leadership Scale. Mil Behav Health.

[12] Skakon J (2010). Are leaders’ wellbeing, behaviours and style associated with the affective wellbeing of their employees? A systematic review of three decades of research. Work Stress.

[13] Clarke S (2013). Safety leadership: A meta-analytic review of transformational and transactional leadership styles as antecedents of safety behaviours. J Occup Organ Psychol.

[14] Adler AB (2014). Behavioral health leadership: new directions in occupational mental health. Curr Psychiatry Rep.

[15] Magnavita N, Tripepi G, Chiorri C (2021). Telecommuting, Off-Time Work, and Intrusive Leadership in Workers' Well-Being. Int J Environ Res Public Health.

[16] Hammer LB (2019). Supervisor support training effects on veteran health and work outcomes in the civilian workplace. J Appl Psychol.

[17] Adler AB (2021). Sleep leadership in the army: A group randomized trial. Sleep Health.

[18] Adler AB (2017). Professional Stress and Burnout in U.S. Military Medical Personnel Deployed to Afghanistan. Mil Med.

[19] Adler AB (2018). Quarantine and the U.S. military response to the Ebola crisis: soldier health and attitudes. Public Health.

[20] Kessler RC (2014). Thirty-day prevalence of DSM-IV mental disorders among nondeployed soldiers in the US Army: results from the Army Study to Assess Risk and Resilience in Servicemembers (Army STARRS). JAMA Psychiat.

[21] Kroenke K, Spitzer RL, Williams JBW (2003). The Patient Health Questionnaire-2 - Validity of a two-item depression screener. Med Care.

[22] Kroenke K (2007). Anxiety disorders in primary care: prevalence, impairment, comorbidity, and detection. Ann Intern Med.

[23] Kroenke K, Spitzer RL, Williams JB (2001). The PHQ-9: validity of a brief depression severity measure. J Gen Intern Med.

[24] Cates D (2021). Minimizing psychological distress and promoting resilience during quarantine: Piloting the town hall model. Prof Psychol Res Pract.

[25] Meadows SO (2018). 2015 Department of Defense Health Related Behaviors Survey (HRBS). Rand Health Q.

[26] Levin-Rector A (2018). Predictors of posttraumatic stress disorder, anxiety disorders, depressive disorders, and any mental health condition among U.S. Soldiers and Marines, 2001–2011. J Trauma Stress.

[27] Nelson BW (2020). Rapid assessment of psychological and epidemiological correlates of COVID-19 concern, financial strain, and health-related behavior change in a large online sample. PLoS ONE.

[28] Ragins B (1989). Power and Gender Congruency Effects in Evaluations of Male and Female Managers. J Manag.

[29] Magnavita N, Tripepi G, Di Prinzio RR (2020). Symptoms in Health Care Workers during the COVID-19 Epidemic. A Cross-Sectional Survey. Int J Environ Res Public Health.

[30] Chirico F (2021). Prevalence of anxiety, depression, burnout syndrome, and mental health disorders among healthcare workers during the COVID-19 pandemic: A rapid umbrella review of systematic reviews. J Health Soc Sci.

[31] R Core Team R (2021). A language and environment for statistical computing.

[32] Military OneSource (2020). 2019 Demographic Profile.

[33] Beymer MR (2021). Association between Food Insecurity, Mental Health, and Intentions to Leave the US Army in a Cross-Sectional Sample of US Soldiers. J Nutr.

[34] Badr H (2021). Sociodemographic and Health Belief Model Factors Associated with Nonadherence to COVID-19 Mitigation Strategies in the United States. Ann Behav Med.

[35] Resnicow K (2021). Novel Predictors of COVID-19 Protective Behaviors Among US Adults: Cross-sectional Survey. J Med Internet Res.

[36] Smith RA (2021). Exploring Behavioral Typologies to Inform COVID-19 Health Campaigns: A Person-Centered Approach. J Health Commun.

[37] Buhler A, Willmund GD (2021). Adherence and Psychosocial Well-Being During Pandemic-Associated Pre-deployment Quarantine. Front Public Health.

[38] Chirico F, Ferrari G (2021). Role of the workplace in implementing mental health interventions for high-risk groups among the working age population after the COVID-19 pandemic. J Health Soc Sci.

